# Integrative data of a novel ciliate (Alveolata, Ciliophora) propose the establishment of *Heterodeviata nantongensis* nov. sp.

**DOI:** 10.1186/s12866-024-03190-y

**Published:** 2024-01-19

**Authors:** Lijian Liao, Limin Jiang, Xiaozhong Hu

**Affiliations:** 1 College of Fisheries, & Key Laboratory of Evolution and Marine Biodiversity of Ministry of Education, Ocean University of China, Qingdao, 266003 China; 2 Institute of Evolution and Marine Biodiversity, Ocean University of China, Qingdao, 266003 China

**Keywords:** Brackish water, Deviatidae, Infraciliature, SSU rRNA gene, Taxonomy

## Abstract

**Background:**

As unicellular eukaryotes, ciliates are an indispensable component of micro-ecosystems that play the role of intermediate nutrition link between bacteria or algae and meiofauna. Recent faunistic studies have revealed many new taxa of hypotrich ciliates, indicating their diversity is greater than previously thought. Here we document an undescribed form isolated from an artificial brackish water pond in East China. Examination of its morphology, ontogenesis and molecular phylogeny suggests that it represents a new species.

**Results:**

The morphology and morphogenesis of the new brackish-water deviatid ciliate, *Heterodeviata nantongensis* nov. sp., isolated from Nantong, China, were investigated using live observations and protargol staining. The diagnostic traits of the new species include three frontal cirri, one buccal cirrus, one or two parabuccal cirri, an inconspicuous frontoventral cirral row of four to six frontoventral cirri derived from two anlagen, three left and two right marginal rows, two dorsal kineties, dorsal kinety 1 with 9–14 dikinetids and dorsal kinety 2 with only two dikinetids, and one to three caudal cirri at the rear end of dorsal kinety 1. Its main morphogenetic features are: (i) the old oral apparatus is completely inherited by the proter except undulating membranes, which are reorganized in situ; (ii) anlagen for marginal rows and the left dorsal kinety develop intrakinetally in both proter and opisthe; (iii) dorsal kinety 2 is generated dorsomarginally; (iv) five cirral anlagen are formed in both proter and opisthe; (v) in the proter, anlagen I and II very likely originate from the parental undulating membranes and the buccal cirrus, respectively, anlage III from anterior parabuccal cirrus, anlage IV originates from the parental frontoventral cirri and anlage V from the innermost parental right marginal row; and (vi) anlagen I–IV of the opisthe are all generated from oral primordium, anlage V from the innermost parental right marginal row. Phylogenetic analyses based on SSU rRNA gene sequence data were performed to determine the systematic position of the new taxon.

**Conclusions:**

The study on the morphology, and ontogenesis of a new brackish-water taxon increases the overall knowledge about the biodiversity of this ciliate group. It also adds to the genetic data available and further provides a reliable reference for environmental monitoring and resource investigations.

## Background

Ciliated protozoans, as a large group of complex and highly differentiated unicellular eukaryotes, have an extremely high species diversity currently with more than ten thousand nominal species [e.g., 1–20]. It is a great challenge to accurately identify and separate these species. However, with the employment of the integrative techniques including protargol staining and gene sequencing, the situation mentioned above has been improved [21–30]. Hypotrichia Stein, 1859, most members of which present a dorsoventrally flattened body shape with prominent cirri on the ventral side and inconspicuous bristles on the dorsal side, and live as benthic forms, is considered to be the most complex and highly differentiated ciliate group. Though hypotrichs have attracted extensive attention, especially in recent years, it remains one of the most confused groups in terms of their systematics [31–40].

Foissner (2016) established a new family Deviatidae to comprise *Deviata* Eigner, 1995, *Notodeviata* Foissner, 2016, and *Idiodeviata* Foissner, 2016 [7, 41]. Very recently, Gao et al. assigned the genera *Pseudosincirra* and *Perisincirra* to the family, and emended the diagnostic traits of this family [26]. Song et al. (2023) further added the genus *Heterodeviata* to the family [21]. These deviatid genera share the two following features: (i) several longitudinal cirral rows dividing intrakinetally or with multiple within-anlagen; and (ii) the absence of transverse cirri. There are a total of fifteen species within the family, though sequence information is only available for nine reported species. Furthermore, both morphogenesis and gene sequences are only available for several species from the genera *Deviata* and *Heterodeviata*. As a result, extensive investigations are needed to elucidate the diversity within this taxon.

During a faunistic study along the coastal area of the Yellow Sea, a *Deviata*-like ciliate was found and subsequent microscopic and phylogenetic investigations suggested it represents an unknown species in the newly erected genus *Heterodeviata* Song et al., 2023.

### ZooBank registration

The ZooBank registration number of present work is: urn:lsid:zoobank.org:pub: 100FD985-06F3-416B-B228-79CADAB7D67D

## Results

### Taxonomy

Class Spirotrichea Bütschli, 1889.

Order Stichotrichida Fauré-Fremiet, 1961.

Family Deviatidae Foissner, 2016.

Genus *Heterodeviata* Song et al., 2023.

*Improved diagnosis*. Medium-sized dorsomarginalian Deviatidae with frontal, buccal and caudal cirri; oral primordium originates apokinetally between right and left cirral rows; two or more left and right marginal rows each, one frontoventral cirral row; four or five frontoventral cirral anlagen, dorsal kinety 2 originates dorsomarginally.

#### ***Heterodeviata nantongensis *nov. sp.**

##### ***ZooBank registration***

*Heterodeviata nantongensis* nov. sp.: urn:lsid:zoobank.org:act: 9FBCC5D9-FF09-4186-90E4-ADA77F06C5EB

*Diagnosis*. Brackish water species with a size of 190–275 × 30–50 μm in vivo and an elongated elliptical body shape; contractile vacuole at midline slightly below mid-body; about four macronuclear nodules and two micronuclei. Adoral zone extending about 19% of body length in vivo, composed of on average 19 membranelles; paroral and endoral membrane straight and optically parallel to each other but staggered by 25%. Three frontal cirri, one buccal cirrus, one or two parabuccal cirri, and an inconspicuous frontoventral cirral row of four to six frontoventral cirri, three left and two right marginal rows; two dorsal kineties; kinety 1 bipolar with one to three caudal cirri at its rear end, kinety 2 reduced to two subapical bristles.

**Fig. 1:**
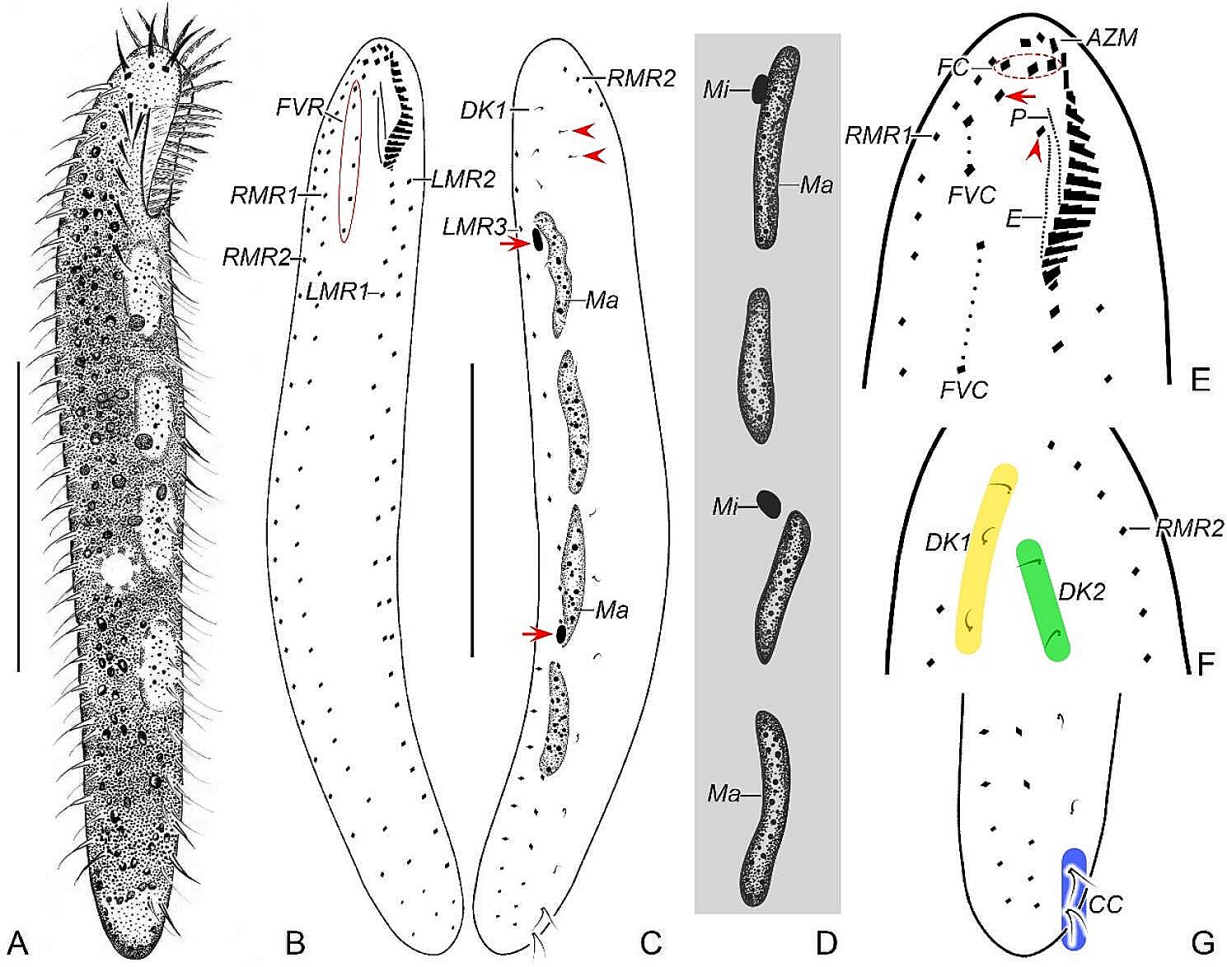
*Heterodeviata nantongensis* nov. sp. from life (**A**) and after protargol staining (**B**–**G**). **A** Ventral view of a representative individual. **B**, **C** Ventral (**B**) and dorsal (**C**) view of the holotype, showing the infraciliature and nuclear apparatus, arrows and arrowheads in (**C**) denote the micronuclei and two bristles of dorsal kinety 2, separately. **D** Macronuclear nodules and micronuclei. **E** Detailed ventral view of the anterior region, arrow shows the parabuccal cirrus and arrowhead marks the buccal cirrus. **F** Detailed dorsal view of the anterior region. **G** Detailed dorsal view of the posterior region. AZM, adoral zone of membranes; CC, caudal cirri; DK1, 2, dorsal kinety 1, 2; E, endoral membrane; FC, frontal cirri; FVC, frontoventral cirri; FVR, frontoventral cirral row; LMR1, 2, 3, left marginal row 1, 2, 3; Ma, macronuclear nodules; Mi, micronuclei; P, paroral membrane; RMR1, 2, right marginal row 1, 2. Scale bars = 80 μm (**A**), 70 μm (**B**, **C**)

**Fig. 2:**
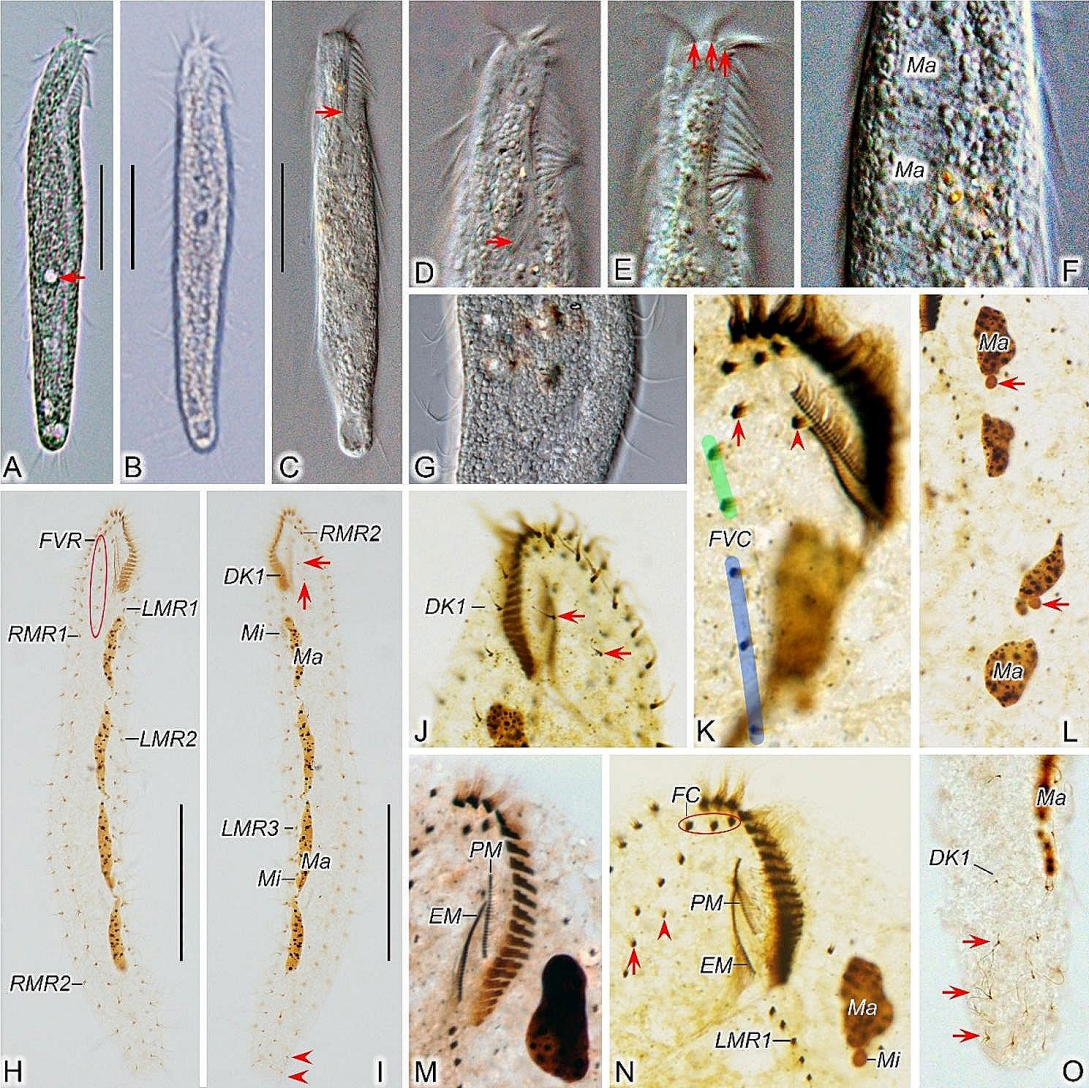
**A**–**O** Morphology of *Heterodeviata nantongensis* nov. sp. from life (**A**–**G**) and after protargol staining (**H**–**O**). **A**–**C** Ventral view of a representative individual, arrow in (**A**) indicates the contractile vacuole and arrow in (**C**) showing buccal lip. **D**, **E** Detail of the anterior end of body, arrow in (**D**) indicates pharyngeal fibers and arrows in (**E**) indicate frontal cirri. **F** Showing the cytoplasm and macronuclear nodules. **G** Showing granular inclusions and marginal cirri. **H**, **I** Ventral (**H**) and dorsal (**I**) views of the holotype, denoting infraciliature and nuclear apparatus, arrows show the two dorsal bristles of dorsal kinety 2 and arrowheads denote the caudal cirri. **J** Dorsal view of the anterior portion of cell, showing the left dorsal kinety and dorsomarginal kinety composed of two dikinetids (arrows). **K** Details of the oral zone, arrow indicates the parabuccal cirrus and arrowhead marks the buccal cirrus. **L** Showing the nuclear apparatus, arrows indicate micronuclei. **M** Details of paroral membrane and endoral membrane. **N** Arrowhead indicates anterior segment of frontoventral row and arrow indicates posterior segment in a daughter cell just after division. **O** Showing three caudal cirri behind the dorsal kinety 1, arrows indicate the caudal cirri. DK1, dorsal kinety 1; EM, endoral membrane; FC, frontal cirri; FVR, frontoventral cirral row; LMR1, 2, 3, left marginal row 1, 2, 3; Ma, macronuclear nodules; Mi, micronuclei; PM, paroral membrane; RMR1, 2, right marginal row 1, 2; Scale bars = 60 μm

*Type specimens*. One slide (registration number: LLJ2020122601/1) containing the holotype specimen (circled with black ink on the back of the slide; Figs. 1B and C and 2H and I) and nineteen paratype slides (registration numbers: LLJ2020122601/2–19) with protargol-stained morphostatic and dividing specimens have been deposited in the Laboratory of Systematic Taxonomy, Ocean University of China, China. Another slide with protargol-stained specimens (registration number: LLJ2020122601/20) has been deposited in the Marine Biological Museum, Chinese Academy of Sciences, Qingdao, China.

*Type locality*. An artificial brackish water pond (31°43′47′′N, 121°56′53′′E), Nantong, China.

*Etymology*. The species-group name *nantongensis* (Latin adjective; originating from Nantong) means the species was first discovered in Nantong, China.

*Ecology*. The water temperature and salinity at the collection site were 6 °C and 8‰, respectively. The new species is a very voracious consumer, and grows well at room temperature in Petri dishes with brackish water (salinity 12‰) to which a few rice grains were added to stimulate the growth of bacteria.

*Morphological description*. Cell size 190–275 × 30–50 μm in vivo (*n* = 6), and 150–250 × 25–80 μm after protargol staining (Table 1), with length: width ratio about 6.2:1 in live state (in a range 4.9–8.0:1). Body flexible but non-contractile, elongated elliptical, with both ends slightly narrowed and rounded (Figs. 1A and 2A–C). Mostly four, rarely five (3 of 17 specimens examined) macronuclear nodules situated left of midline. Consistently two oval micronuclei (about 3.0 μm in diameter) observed in protargol preparations but difficult to recognize in vivo; one attached to the first macronuclear nodule (counted posteriad) and the other very near the third macronuclear nodule (Figs. 1C and D and 2F, I, L and N). Contractile vacuole (about 9.0 μm in diameter) located at midline slightly below mid-body (Fig. 2A). Locomotion slowly, usually gliding over impurities on surface of Petri dishes with body obviously twisted.

**Table 1:** Morphometrical characterization of *Heterodeviata nantongensis* nov. sp.

Character	Min	Max	Mean	M	SD	CV	SEM	n
Body length	149	246	198.9	204	34.4	17.3	8.1	18
Body width	24	78	51.0	51	14.9	29.2	3.5	18
Body length:width, ratio	2.7	6.9	4.2	3.7	1.3	30.5	0.3	18
Length of AZM	24	34	28.0	28	3.2	11.4	0.8	18
AZM length: body length, ratio	0.11	0.21	0.15	0.15	0.03	0.22	0.008	18
Adoral membranelles, number	17	20	18.5	19	0.9	4.6	0.2	18
Macronuclear nodules, number	4	5	4.2	4	0.4	10.1	0.1	18
Macronuclear nodules, length in µm	14	36	21.1	20	5.7	27.3	1.4	18
Macronuclear nodules, width in µm	3	9	5.3	5	1.7	32.3	0.4	18
Micronuclei, number	2	2	2.0	2	0	0	0	18
Micronuclei, diameter in µm	2	5	3.0	3	0.7	24.0	0.2	18
Frontal cirri, number	3	3	3.0	3	0	0	0	18
Buccal cirrus, number	1	1	1.0	1	0	0	0	18
Parabuccal cirri, number	1	2	1.1	1	0.3	29.1	0.1	18
Frontoventral cirri, number	4	6	4.5	4	0.7	15.7	0.2	18
Anterior body end to end of FVR, distance	28	62	40	38	8.4	20.9	1.8	21
Left marginal rows, number	3	3	3.0	3	0	0	0	18
Cirri in LMR 1, number	15	22	18.6	19	1.9	10.3	0.5	18
Cirri in LMR 2, number	14	20	16.4	17	1.3	8.1	0.3	18
Cirri in LMR 3, number	13	20	15.6	15	1.7	10.8	0.4	18
Right marginal rows, number	2	2	2	2	0	0	0	18
Cirri in RMR 1, number	20	31	25.3	25	3.1	12.3	0.7	18
Cirri in RMR 2, number	20	30	24.9	25	2.7	11.0	0.6	18
Dorsal kineties, number	2	2	2	2	0	0	0	18
Dikinetids in dorsal kinety 1, number	9	14	11.6	11	1.5	12.6	0.3	18
Dikinetids in dorsal kinety 2, number	2	2	2	2	0	0	0	18
Caudal cirri, number	1	3	1.7	2	0.6	35.6	0.1	18

All measurements are in micrometres. All data are based on protargol-stained specimens. AZM, adoral zone of membranelles; CV, coefficient of variation in %; FVR, frontoventral cirral row; LMR, left marginal row; M, median; Max, maximum; Mean, arithmetic mean; Min, minimum; n, number of specimens measured; RMR, right marginal row; SD, standard deviation; SEM, standard error of mean

Buccal field narrow and inconspicuous, occupying about 19% of body length in vivo and on average 15% of body length (in a range of 11–21%) after protargol staining (Figs. 1A and B and 2A, C and H). Adoral zone of membranelles mainly running along left anterior body margin with proximal portion slightly extending towards cell midline, composed of 17–20 membranelles. Paroral and endoral membranes almost straight, nearly the same length and both generally single-rowed, optically side by side but staggered by 25% (Figs. 1E and 2K, M and N). Pharyngeal fibers conspicuous, about 35 μm long (Fig. 2D and N). Constantly three slightly enlarged frontal cirri; one buccal cirrus right of anterior of paroral membrane. One or two parabuccal cirri behind the rightmost frontal cirrus (Figs. 1E and 2E and K). An inconspicuous frontoventral cirral row of four to six frontoventral cirri, arranged in two segments, the posterior one slightly dislocated to the left of the anterior one for most morphostatic cells (Figs. 1B and E and 2H and K), but distinctly displaced to the right in daughter cell just after division (Fig. 2N). Consistently three left marginal rows, the innermost and outermost row with 15–22 and 13–20 cirri, respectively, the middle row with 14–20 cirri; two right marginal rows, each with about 25 cirri (Figs. 1B and C and 2G, H and I). Marginal cirri roughly 13 μm long in protargol-stained specimens. Frontoventral cirri and several anterior cirri in each marginal row usually slightly thickened, i.e., composed of 6–8 cilia; main portion of marginal rows with cirri composed of four cilia; most cirri at rear portion composed of only two cilia (Figs. 1B and C and 2H, I and L).

Invariably two dorsal kineties, the left kinety bipolar, composed of 9–14 dikinetids, the right one (dorsomarginal kinety) highly reduced, with only two dikinetids; only anterior kinetid bearing a bristle about 3 μm long in stained cells. One to three caudal cirri located at the end of the left kinety, consisting of two kinetids with each bearing a probably 15 μm long cilium (Figs. 1C, F and G and 2I, J and O).

### Morphogenesis during binary division

#### Stomatogenesis

**Fig. 3:**
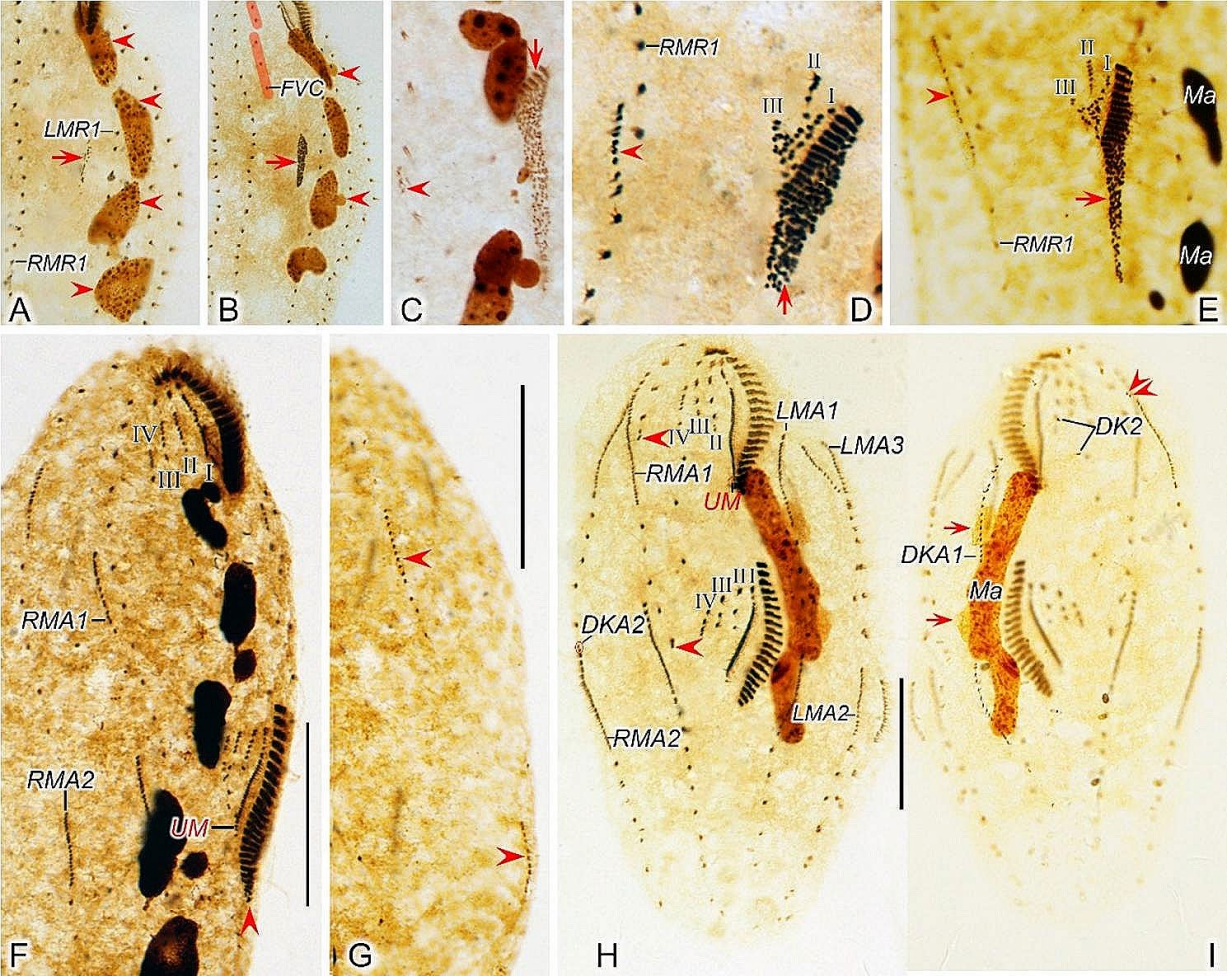
Early to middle morphogenetic stages of *Heterodeviata nantongensis* nov. sp. after protargol staining. **A** Ventral view of very early dividers, arrow showing oral primordium of the opisthe and arrowheads showing macronuclear nodules. **B** Arrowheads indicate micronuclei and arrow denotes oral primordium of the opisthe. **C** Ventral view of an early divider, arrowhead showing the intrakinetally formed marginal anlagen and arrow indicates several newly formed adoral zone of membranes. **D**, **E** Ventral views of early divider, arrowheads and arrows in (**D**, **E**) separately indicate the marginal anlagen and developed oral primordia. **F, G** Ventral (**F**) and dorsal (**G**) view of the same early divider, arrowhead in (**F**) indicates incompletely formed adoral zone of membranes and arrowheads in (**G**) showing the dorsal kinety anlage 1, in this stage, anlagen I–IV of the proter and opisthe formed. **H, I** Ventral (**H**) and dorsal (**I**) view of the same early-middle divider, arrowheads in (**H**) showing the freshly formed anlage V of the proter and opisthe, arrowheads and arrows in (**I**) showing the dorsal kinety anlage 2 develops from the proximal end of RMA2 and the micronuclei, separately. I–IV, anlagen I–IV; DKA1, 2, dorsal kinety anlagen 1, 2; FVC, frontoventral cirri; LMA1, 2, 3, left marginal anlagen 1, 2, 3; LMR1, left marginal row 1; Ma, macronuclear nodules; RMA1, 2, right marginal anlagen 1, 2; RMR1, right marginal row 1; UM, undulating membrane. Scale bars = 35 μm (**F**, **G**), 30 μm (**H, I**)

**Fig. 4:**
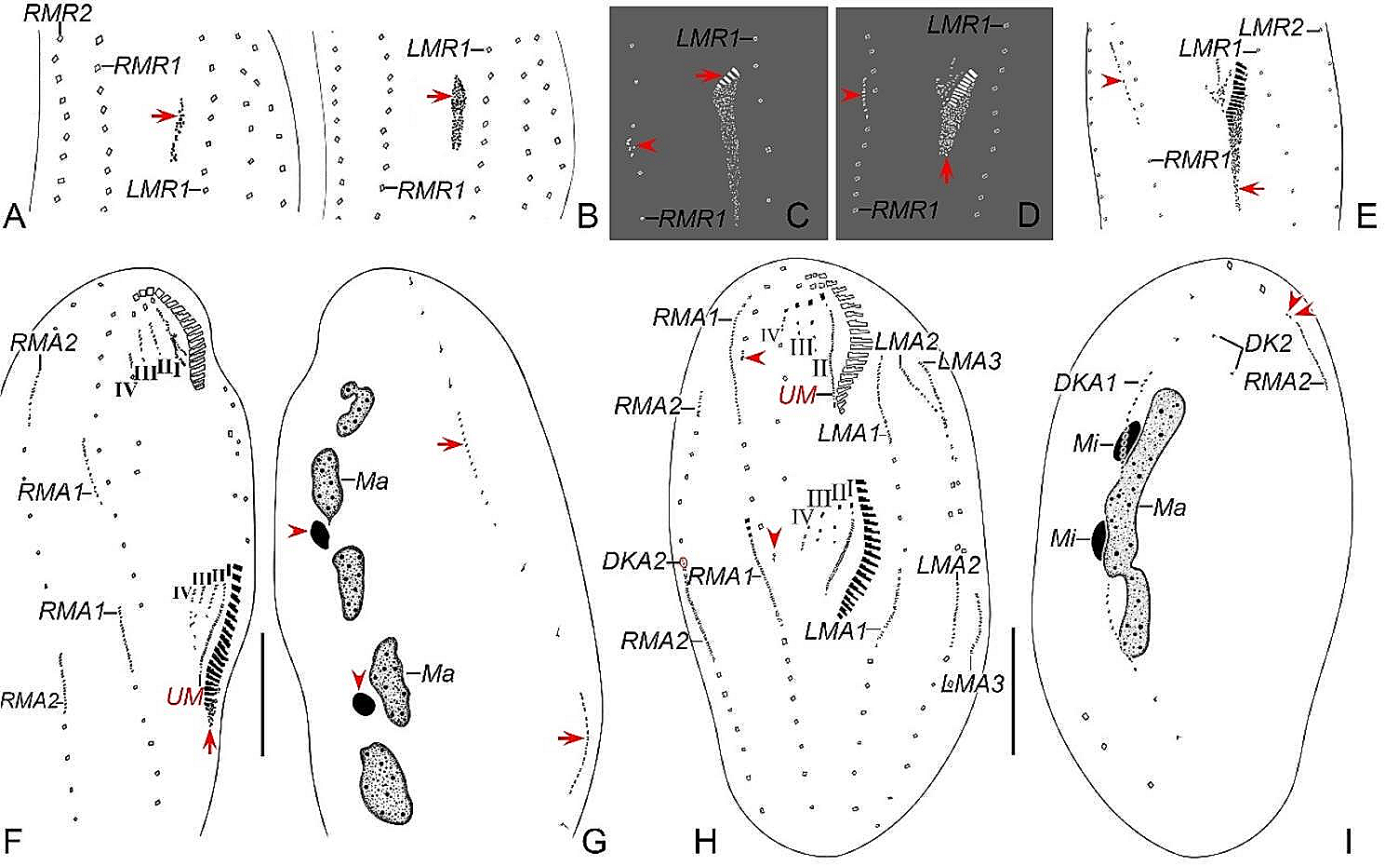
Early to middle morphogenetic stages of *Heterodeviata nantongensis* nov. sp. after protargol staining. **A**, **B** Ventral view of very early dividers, arrows in (**A**, **B**) showing oral primordium of the opisthe. **C** Ventral view of an early divider, arrowhead indicates the intrakinetally formed marginal anlagen and arrow denotes several newly formed adoral zone of membranes. **D**, **E** Ventral views of early divider, arrowheads and arrows in (**D**, **E**) separately indicate the marginal anlagen and developed oral primordia. **F, G** Ventral (**F**) and dorsal (**G**)view of the same early divider, arrow in (**F**) indicates uncompletely formed adoral zone of membranes, arrows and arrowheads in (**G**) showing the dorsal kinety anlage 1 and micronuclei, in this stage, anlagen I–IV of the proter and opisthe formed. **H, I** Ventral (**H**) and dorsal (**I**)view of the same early-middle divider, arrowheads in (**H**) showing the freshly formed anlage V of the proter and opisthe, arrowheads in (**I**) showing the dorsal kinety anlage 2 develops from the proximal end of RMA2. I–IV, anlagen I–IV; DKA1, 2, dorsal kinety anlagen 1, 2; LMR1, 2, left marginal row 1, 2; LMA1, 2, 3, left marginal anlagen 1, 2, 3; Ma, macronuclear nodules; Mi, micronuclei; RMR1, 2, right marginal row 1, 2; RMA1, 2, right marginal anlagen 1, 2; UM, undulating membrane. Scale bars = 30 μm

**Fig. 5:**
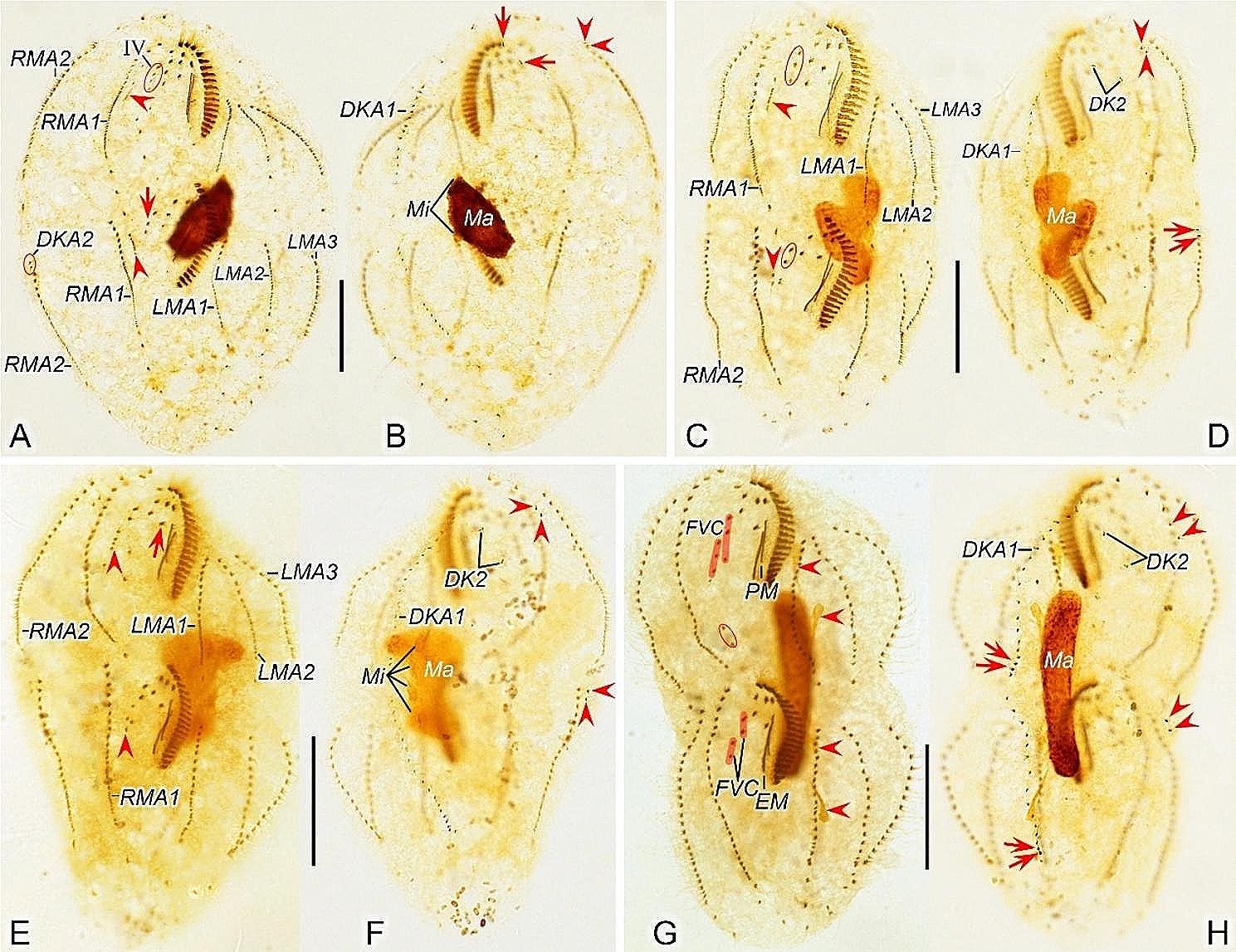
Middle to late morphogenetic stages of *Heterodeviata nantongensis* nov. sp. after protargol staining. **A**, **B** Ventral (**A**) and dorsal (**B**) view of the same middle divider, arrow in (**A**) indicates anlage IV of the opisthe and arrowheads in (**A**) denote the anlage V, arrowheads in (**B**) indicate the dorsal kinety anlage 2 of the proter and arrows in (**B**) showing the old dorsal kinety, in this stage, the macronuclear nodules fuse into a single mass. **C**, **D** Ventral (**C**) and dorsal (**D**) view of the same middle-late divider, arrowheads in (**C**) indicate the anlage V and ellipses encircle the anlage IV of the proter and opisthe, arrowheads and arrows in (**D**) indicate the dorsal kinety anlage 2 of the proter and opisthe, separately, in this stage, dorsal kinety anlage 2 of the proter migrates rightward and appears to the right of new right marginal row 2. **E**, **F** Ventral (**E**) and dorsal (**F**) view of the same middle-late divider, arrow indicates buccal cirrus and arrowhead in (**E**) showing anlage V migrates leftward and arrowheads in (**F**) indicate the dorsal kinety anlage 2 of the proter and opisthe, in this stage, dorsal kinety anlage 2 of the opisthe migrates rightward and appears to the right of new right marginal row 2. **G**, **H** Ventral (**G**) and dorsal (**H**) view of the same middle-late divider, ellipse in (**G**) encircles the parental marginal cirri and arrowheads in (**G**) indicate the micronuclei, and arrows in (**H**) indicate caudal cirrus for the proter and opisthe and arrowheads in (**H**) denote dorsal kinety anlage 2. IV, anlage IV; DK2, dorsal kinety 2; DKA1, 2, dorsal kinety anlagen 1, 2; EM, endoral membrane; FVC, frontoventral cirri; LMA1, 2, 3 left marginal anlagen 1, 2, 3; Ma, macronuclear nodules; Mi, micronuclei; PM, paroral membrane; RMA1, 2, right marginal anlagen 1, 2. Scale bars = 30 μm

**Fig. 6:**
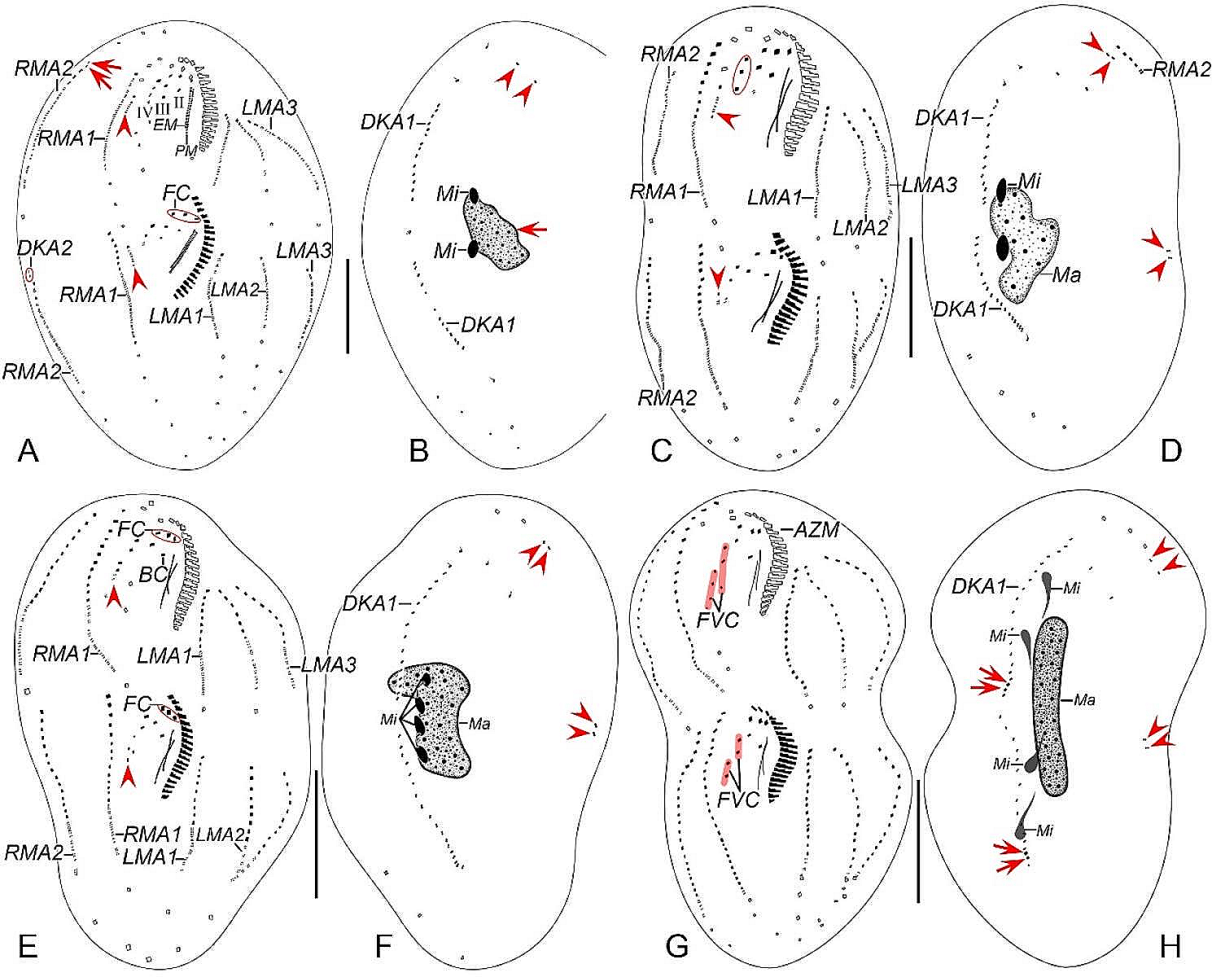
Middle to late morphogenetic stages of *Heterodeviata nantongensis* nov. sp. after protargol staining. **A**, **B** Ventral (**A**) and dorsal (**B**) view of the same middle divider, arrowheads and arrows in (**A**) denote the anlage V and the dorsal kinety anlage 2 of the proter, and arrowheads and arrow in (**B**) indicate dorsal kinety 2 and macronuclear nodules, separately. **C**, **D** Ventral (**C**) and dorsal (**D**) view of the same middle-late divider, arrowheads in (**C**) indicate the anlage V, ellipse in (**C**) encircles the anlage IV of the proter and arrowheads in (**D**) indicate the dorsal kinety anlage 2 of the proter and opisthe. **E**, **F** Ventral (**E**) and dorsal (**F**) view of the same middle-late divider, arrowheads in (E) denote the anlage V of the proter and opisthe, arrowheads in (**F**) indicate the dorsal kinety anlage 2 of the proter and opisthe, in this stage, the micronuclei divide mitotically once. **G**, **H** Ventral (**G**) and dorsal (**H**) view of the same middle-late divider, arrows and arrowheads in (**H**) indicate caudal cirri and dorsal kinety anlage 2 for the proter and opisthe, in this stage, anlagen I–V dedifferentiated into cirri. I–IV, anlagen I–IV; AZM, adoral zone of membranes; BC, buccal cirrus; DKA1, 2, dorsal kinety anlagen 1, 2; EM, endoral membrane; FC, frontal cirri; FVC, frontoventral cirri; LMA1, 2, 3 left marginal anlagen 1, 2, 3; Ma, macronuclear nodules; Mi, micronuclei; PM, paroral membrane; RMA1, 2, right marginal anlagen 1, 2. Scale bars = 30 μm

In early dividers, stomatogenesis commences with the de novo formation of opisthe’s oral primordium (OP) at the middle portion of cell between the innermost right and left marginal rows (Figs. 3A and 4A). With the proliferation of basal bodies, the OP enlarges and several membranelles are organized in its anterior portion; meanwhile, undulating membranes anlage (anlage I) is detached from the right of oral primordium (Figs. 3B–E and 4B–E). With further development, the formation of all new membranelles are gradually completed (Figs. 3H and 4H); the posterior majority of anlage I split longitudinally to form paroral and endoral membrane (Figs. 3F and H, 4F and H, 5A and 6A). The parental adoral zone of membranelles remains intact and is wholly inherited by the proter (Figs. 3F and H and 4F and H). In early dividers, the old undulating membranes dedifferentiate in situ and forms anlage I for the proter, which then develop in the same way as in opisthe (Figs. 3H and I and 4H and I).

#### Ventral ciliature

**Fig. 7:**
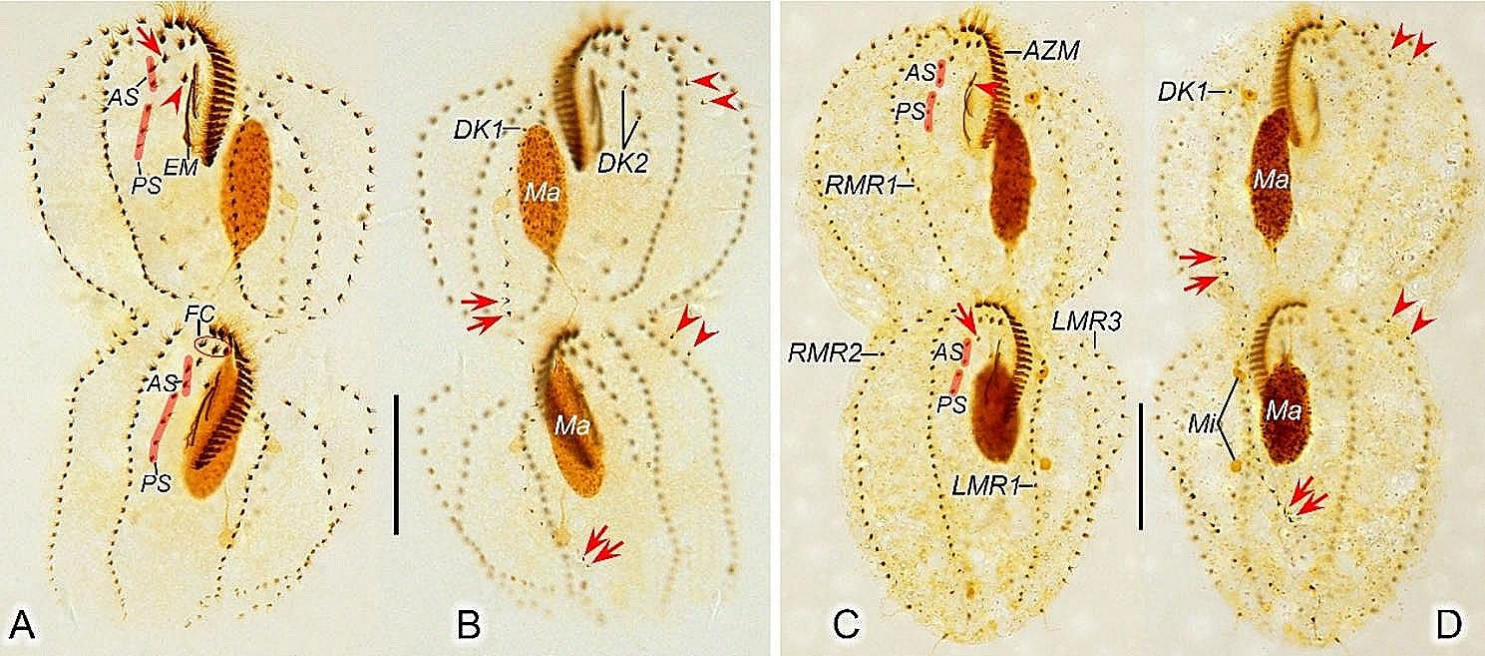
Late morphogenetic stages of *Heterodeviata nantongensis* nov. sp. after protargol staining. **A**, **B** Ventral (**A**) and dorsal (**B**) view of the same late divider, arrow and arrowhead in (**A**) indicate parabuccal cirrus and buccal cirrus of the proter, arrows in (**B**) indicate the caudal cirri of the proter and opisthe and arrowheads in (**B**) denote the dorsal kinety 2, in this stage, two bristles of dorsal kinety 2 formed and one macronuclear nodule dedifferentiated into two ones. **C**, **D** Ventral (**C**) and dorsal (**D**) view of the same late divider, arrowhead in (**C**) indicates the buccal cirrus of the proter and arrow in (**C**) indicates the parabuccal cirrus of the opisthe, and arrows and arrowheads in (**D**) denote the caudal cirri and dorsal kinety 2 of the proter and opisthe. AS, anterior segment of frontoventral cirri; AZM, adoral zone of membranes; DK1, 2, dorsal kinety 1, 2; EM, endoral membrane; FC, frontal cirri; LMR1, 3, left marginal row 1, 3; Ma, macronuclear nodules; PS, posterior segment of frontoventral cirri; RMR1, 2, right marginal row 1, 2. Scale bars = 30 μm

**Fig. 8:**
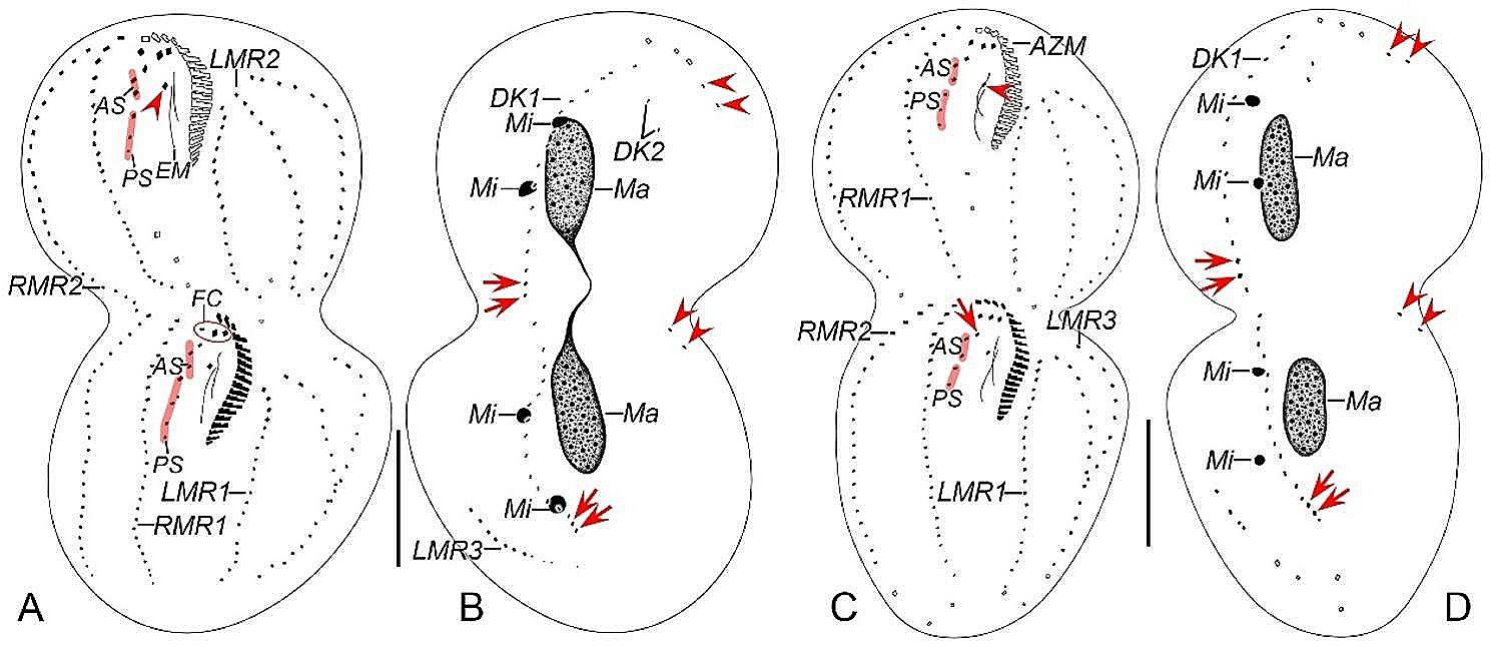
Late morphogenetic stages of *Heterodeviata nantongensis* nov. sp. after protargol staining. **A**, **B** Ventral (**A**) and dorsal (**B**) view of the same late divider, arrowhead in (**A**) indicates buccal cirrus, arrows and arrowheads in (**B**) indicate the caudal cirri and dorsal kinety 2 of the proter and opisthe. **C**, **D** Ventral (**C**) and dorsal (**D**) view of the same late divider, arrowhead in (**C**) indicates the buccal cirrus of the proter and arrow in (**C**) indicates the parabuccal cirrus of the opisthe, and arrows and arrowheads in (**D**) denote the caudal cirri and dorsal kinety 2 of the proter and opisthe, separately. AS, anterior segment of frontoventral cirri; AZM, adoral zone of membranes; DK1, 2, dorsal kinety 1, 2; EM, endoral membrane; FC, frontal cirri; LMR1, 2, 3, left marginal row 1, 2, 3; Ma, macronuclear nodules; Mi, micronuclei; PS, posterior segment of frontoventral cirri; RMR1, 2, right marginal row 1, 2. Scale bars = 30 μm

Cirral anlagen II–V evidently appear later than OP. They originate earlier in the opisthe than in the proter. In opisthe, anlagen II–IV originate from the OP; whereas in proter, they are derived from the dedifferentiation of buccal cirrus, parabuccal cirrus and anterior frontoventral cirri, respectively according to their positions (Figs. 3F and H and 4F and H). After the completion of the formation of the new adoral zone of membranelles, anlage V begins to appear within the innermost right marginal row (RMR1) in both proter and opisthe and then migrates leftward (Figs. 3H, 4H, 5A, C and E and 6A, C and E). These anlagen plus small anterior part of anlage I (as mentioned above) gradually differentiate into new cirri (Figs. 3H, 4H, 5A and 6A). Anlage I generates the left frontal cirrus. Anlage II forms the middle frontal cirrus and buccal cirrus. Anlage III produces the right frontal cirrus and mostly one parabuccal cirrus. Anlagen IV and V form two or three and two to four frontoventral cirri, respectively (Figs. 5E and G, 6E and G, 7A and C and 8A and C).

Likewise, marginal cirral anlagen also appear later in the proter than in the opisthe. Most anlagen develop intrakinetally within parental rows except majority of the anlage for the innermost marginal row (RMA1), which are mostly formed de novo right of the old row (Figs. 3C and 4C). It is evident that only few old cirri in the innermost marginal row dedifferentiate and contribute to the formation of the RMA1. With further proliferation of basal bodies, these anlagen lengthen towards both ends and produce new cirri to replace the old ones at the final stage of division (Figs. 3D–F and H, 4 D–F and H, 5A, C, E and G, 6A, C, E and G, 7A and C and 8A and C).

#### Dorsal ciliature

In early dividers, dorsal kineties anlagen (DKA1) are formed intrakinetally within the parental kinety at two levels corresponding to proter and opisthe (Figs. 3G and 4G). With the proliferation of basal bodies, DKA1 extend toward both ends to replace the old structures (Figs. 3I, 4I, 5B, D, F and H, 6B, D, F and H, 7B and D and 8B and D). In early to middle dividers, the dorsomarginal kinety anlage (DKA2) containing only two dikinetids develops from the anterior end of the anlage for the outmost right marginal row (RMA2) (Figs. 3H and I, 4H and I, 5A and B and 6A and B). In middle to late dividers, DKA2 migrates leftward and develops into the new DK2, which is finally located between of the new right marginal row 2 and dorsal kinety 1 (Figs. 5D, F and H and 6D, F and H).

#### Nuclear apparatus

In early dividers, DNA synthesis occurs in macronucleus, represented by a replication band in each macronuclear nodule (Fig. 3A and B). In middle dividers, the macronuclear nodules move towards the center of cell and gradually fuse into a singular mass (Figs. 3I, 4I, 5A and B and 6B). During later stages of morphogenesis, this mass divides twice before cytokinesis to generate enough macronuclear nodules for both proter and opisthe (Figs. 5D, F and H, 6D, F and H, 7B and D and 8B and D). The micronuclei divide mitotically (Figs. 3I, 4I, 5G, 6D, F and H, 7B and D and 8B and D).

### SSU rRNA gene sequence and phylogenetic analyses (Fig. 9)

**Fig. 9:**
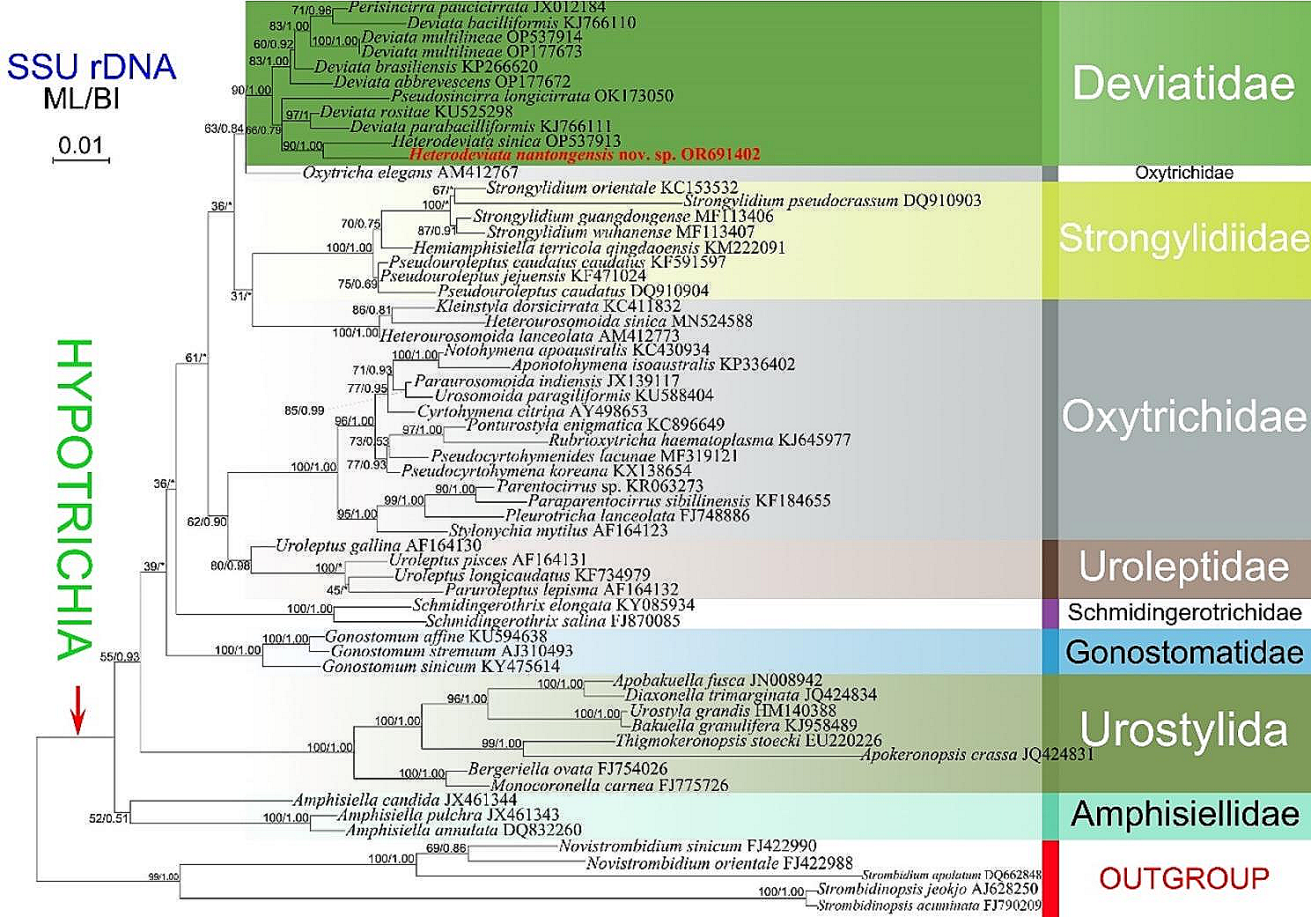
Maximum likelihood (ML) tree based on the SSU rRNA gene sequence data. The newly obtained sequence, *Heterodeviata nantongensis* nov. sp., is indicated in red. Numbers at the nodes represent the ML bootstrap support and BI posterior probability values. Symbol “Asterisks” indicate disagreement between the BI tree and the reference ML tree. All branches are drawn to scale. The scale bar corresponds to one substitution per 100 nucleotide positions

The small subunit ribosomal RNA (SSU rRNA) gene sequence of the new species obtained in this study has been deposited in GenBank under the accession number OR691402. As 1646 base pairs in length, the sequence has a DNA guanine-cytosine (G + C) content of 45.50%.

The phylogenetic trees constructed based on the SSU rRNA gene sequence data using BI and ML analyses are almost congruent, although with some variations in support values between methods. Therefore, only the ML tree topology is presented here, with support values from both BI and ML analyses shown (Fig. 9).

In the phylogenetic trees, members of the family Deviatidae with known sequence cluster together with moderate to full support (90% ML, 1.00 BI). Within this cluster, there are two subclades. One subclade consists of *Perisincirra paucicirrata* Foissner et al., 2002, *Deviata bacilliformis* (Gelei, 1954) Eigner, 1995, *D. multilineae* Zhang et al., 2022, *D. brasiliensis* Siqueira-Castro et al., 2009 and *D. abbrevescens* Eigner, 1995. The other subclade is composed of *Pseudosincirra longicirrata* Gao et al., 2021, *Deviata rositae* Küppers et al., 2007, *D. parabacilliformis* Li et al., 2014, *Heterodeviata sinica* and the new species investigated in this study. The sister relationship between *Heterodeviata nantongensis* nov. sp. and *Heterodeviata sinica* is moderately or maximum supported (90% ML, 1.00 BI). In terms of sequence, the new species is distinguished from *H. sinica* by 28 nucleotides (corresponding to 98.1% sequence similarity). Compared to other deviatids, the new species differs by 28–33 nucleotides (corresponding to 98.1–97.8% similarities) from seven species of *Deviata*, 21 nucleotides (corresponding to 98.6% similarity) from *Perisincirra paucicirrata*, and 36 nucleotides (corresponding to 97.6% similarity) from *Pseudosincirra longicirrata*.

## Discussion

### **Establishment of *Heterodeviata nantongensis *nov. sp.**

In view of several longitudinal cirral rows dividing intrakinetally, the lack of transverse cirri, the oral primordium originated apokinetally between right and left cirral rows and five frontoventral cirral anlagen, the species described in this study should be assigned to the family Deviatidae. When compared with all extant deviatid genera, our form can be easily distinguished from *Deviata* Eigner, 1995 and *Idiodeviata* Foissner, 2016 by the presence (vs. absence) of caudal cirri [7, 41], from *Notodeviata* Foissner, 2016 and *Perisincirra paucicirrata* Foissner et al., 2002 by the presence (vs. absence) of dorsomarginal kinety [7, 43] and from *Pseudosincirra* Gao et al., 2021 by the number of bipolar dorsal kineties (one vs. three in *Pseudosincirra*) [26]. The current form undoubtedly belongs to the newly established genus *Heterodeviata* Song et al., 2023. In terms of the presence of the frontal, buccal, parabuccal and caudal cirri as well dorsomarginal kinety, the absence of transverse cirri, and multiple marginal cirri. Until now, there is only *Heterodeviata sinica* (type species) in the genus. Our species can be easily distinguished by the pattern and origin of frontoventral cirri (forming an indistinct row, and from two anlagen vs. a distinct row, and from single anlage in *Heterodeviata sinica*) [21]. Thus, a new species has to be proposed.

### Morphogenetic comparison

Considering the formative modes of frontal ventral cirral anlagen and dorsal kineties, *Heterodeviata nantongensis* nov. sp. should be compared with some similar species that possess three clearly differentiated frontal cirri within the Deviatidae. Till now, morphogenesis of nine species of deviatids have been investigated in detail, i.e., *Deviata abbrevescens*, *D. bacilliformis*, *D. brasiliensis*, *D. parabacilliformis*, *Idiodeviata venezuelensis* Foissner, 2016, *Notodeviata halophila* Foissner, 2016, *Perisincirra paucicirrata*, *Pseudosincirra longicirrata* and *Heterodeviata sinica*. Four *Deviata* species differ from our species by the number of frontoventral cirral anlagen (six vs. five) and the absence (vs. presence) of dorsomarginal kinety anlage [41, 45–47]. *Idiodeviata venezuelensis* can be separated by the dorsal kinety anlage that does not produce caudal cirri at its rear end [7]. *Notodeviata halophila* differs from our species by all new cirri of anlage IV and most cirri of anlagen II and III are resorbed in late and very late dividers, and the absence of dorsomarginal kinety anlage [7]. *Heterodeviata sinica* differs from our species by the number of frontal ventral cirral anlagen and the origin of frontoventral cirral row (as above) [21]. Both *Perisincirra paucicirrata* and *Pseudosincirra longicirrata* share five frontal ventral anlagen with our new species, but *Perisincirra paucicirrata* lacks dorsomarginal kinety anlage [43] and *Pseudosincirra longicirrata* differs by having three dorsal kinety anlagen (vs. one dorsal kinety anlage in our new species) [26]. Therefore, these two species can be easily distinguished from our form.

While in Kahliellidae Tuffrau, 1979, some parental structures are preserved in most species, i.e., *Kahaliella simplex* (Horváth, 1934) Berger, 2011 (parental left marginal rows retained), *Neogeneia hortualis* Eigner, 1995 (parental marginal cirri retained), and *Parakahliella macrostoma* (Foissner, 1982) Berger et al., 1985 (parental dorsal kineties retained), *Heterodeviata nantongensis* nov. sp. does not retain any old structures in interphase cell) [3, 41]. For some cases (e.g. in *Afrokahliella paramacrostoma* Li et al., 2021 and *Fragmocirrus espeletiae* Foissner, 2000), parental structures are not retained, but they can be separated from *Heterodeviata nantongensis* nov. sp. by the generation of *Urosomoida*-patterned dorsal ciliature [44, 48].

### Phylogenetic analyses

*Heterodeviata nantongensis* nov. sp. falls in a clade that includes seven *Deviata* species, *Perisincirra paucicirrata*, *Pseudosincirra longicirrata* and *Heterodeviata sinica*. The close relationship between these eleven species supported by the shared fine cirri, i.e., cirri in the ventral and marginal rows are mostly composed of two or four cilia and cirri are relatively widely spaced within cirral rows, at least one left and one right marginal row, and cirri within all rows relatively widely spaced [21, 26, 42, 43, 46, 47]. Gao et al. (2021) suggested that the genus *Pseudosincirra* and the species *Perisincirra paucicirrata* should also be assigned to Deviatidae, which is supported by the present study. The presence or absence of dorsomarginal kineties and the number of dorsal kineties vary significantly in these Deviatidae species. Foissner (2016) proposed that the dorsomarginal kineties might have evolved several times independently and inferred that Deviatidae is possibly sister to the non-dorsomarginalian family Kahliellidae Tuffrau, 1979. However, this assumption was not confirmed by the present study and recent studies, which demonstrated a closer relationship between Deviatidae and Dorsomarginalia plus *Strongylidium*–*Hemiamphisiella*–*Pseudouroleptus* [21, 22, 26, 46, 47].

In the current phylogenetic analysis, the well grouping of all deviatid species with SSU rRNA gene sequence data available supports the rationality of the established family Deviatidae by Foissner [7] and the monophyly of the family, which is consistent with previous studies [21, 26, 42]. The monophyly of Deviatidae is also supported by the AU test (*p* = 0.985). In our phylogenetic trees, seven species of *Deviata* do not group a single clade, with members of genera *Perisincirra*, *Pseudosincirra*, *Heterodeviata* nested, which suggests that *Deviata* is non-monophyletic. Interestingly, the monophyly of *Deviata* was not rejected by the AU test (*p* = 0.439).

The sister relationship between *Heterodeviata nantongensis* nov. sp. and *Heterodeviata sinica* conforms to their high morphological and morphogenetic similarities [21], for instance, (i) differentiation of frontal, frontoventral, buccal, and caudal cirri; (ii) absence of transverse cirri; (iii) one bipolar dorsal kinety and one reduced dorsomarginal kinety and their origins; (iv) formation mode of new oral apparatus for daughter cells; and (v) proliferation of anlagen within marginal rows. However, their identities of as distinct species are corroborated by sequence divergences and discrepancies in other morphological and morphogenetic traits as discussed above [21].

## Conclusions

In this study, we described a new ciliate, *Heterodeviata nantongensis* nov. sp., discovered from an artificial brackish water habitat in China. This study increases our knowledge about biodiversity and enriches the database of deviatids. The identity of our form as distinct species is supported by evidences from morphology, morphogenesis and molecular sequences. To date, the family Deviatidae comprises five genera with three being monotypic except *Deviata* and *Heterodeviata*, suggesting most novel taxa may have evolved recently. Furthermore, only nine species have reliable molecular data reported within the family. More deviatid species are expected to be discovered to better elucidate the biodiversity and systematics of this group of ciliates.

## Methods

### Sample collection, observations, and identification

**Fig. 10:**
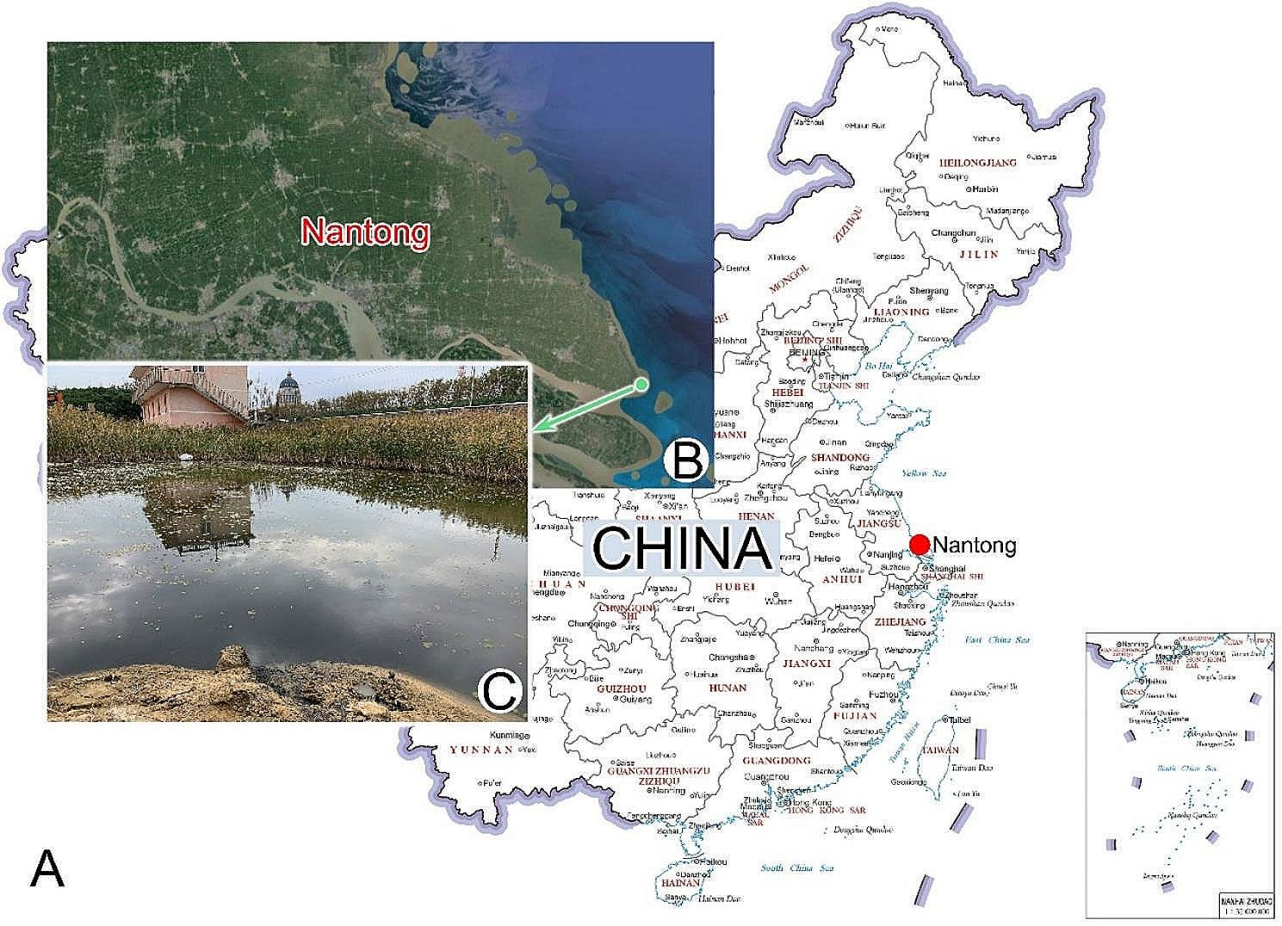
**A**–**C** Locations of the sampling site. **A, B** The map of China from the MAP WORLD (www.tianditu.gov.cn, drawing review number: GS (2019) 1686) (**A**) and portion of Google Map (**B**), showing the location of Nantong, China (32°00′N, 120°53′E). **C** Showing the artificial pond where *Heterodeviata nantongensis* nov. sp. was collected

*Heterodeviata nantongensis* nov. sp. was collected on December 26th 2020 from an artificial brackish water pond (6℃, 8‰) (Fig. 10C) in Nantong, China (Fig. 10A, B). Initial cultures were established by maintaining raw samples at room temperature (approximately 25 °C) in Petri dishes containing habitat pond water. Rice grains were added to the Petri dishes to promote bacterial growth, serving as the food source for the ciliates.

Living cells were observed in vivo under bright field and differential interference contrast illumination using a light microscope (Zeiss AXIO Imager D2 and Olympus BX53). In vivo measurements were conducted at magnification of 40–1,000X. Protargol staining was applied following the method of Wilbert [35] to reveal the infraciliature and nuclear apparatus. Measurements and counts of stained specimens were performed at a magnification of 1,000X. Drawings of stained specimens were done based on micrographs. To illustrate changes during morphogenesis, old (parental) structures are shown outlined, while new ones are shaded black. Classification follows Lynn [15] and Foissner [7], and terminology is according to Berger [3].

### DNA extraction, PCR amplification, and gene sequencing

One to three living cells were isolated from the raw culture and washed five times with sterilized seawater. The cells were then transferred to a 1.5 ml microfuge tube with a minimum volume of water. Genomic DNA was extracted using the DNeasy Blood and Tissue Kit (Qiagen, Germany) according to the manufacturer’s instructions. The SSU rRNA gene was amplified via PCR using the Q5 Hot Start high fidelity DNA polymerase (NEB, USA) and the PCR primers 82 F (5′-GAAACTGCGAATGGCTC-3′) and 18 S-R (5′-TGATCCTTCTGCAGGTTCACCTAC-3′), as described by Medlin et al. [49].

### Phylogenetic analyses

The newly obtained SSU rRNA gene sequence was aligned with 60 other taxa sequences obtained from the GenBank database using MUSCLE on the EBI website (http://www.ebi.ac.uk/Tools/msa/muscle/). Strombidiids including *Novistrombidium orientale* (FJ422988), *Novistrombidium sinicum* (FJ422990), *Strombidium apolatum* (DQ662848), *Strombidinopsis jeokjo* (AJ628250) and *Strombidinopsis acuminate* (FJ790209) were used as outgroup taxa. Accession numbers are provided after species names in the phylogenetic tree. Primer sequences were manually removed from the alignment using Bioedit 7.2.5 according to Hall [50]. Both ends of the alignment were trimmed, resulting in a final refined alignment of taxa with 1752 positions. This alignment was then used to construct the phylogenetic trees.

Maximum likelihood (ML) analysis was performed using IQ-TREE v.2.0 with 10,000 ultrafast bootstrap replicates. The TIM2 + F + R3 model was seleted as the best-fit model according to the Bayesian information criterion (BIC) [51]. Bayesian inference (BI) analysis was conducted using MrBayes 3.2.6 on the CIPRES Science Gateway (XSEDE v.3.2.6) [52], with the GTR + I + G model chosen by Akaike Information Criterion in MrModeltest v2.2 [53]. Markov chain Monte Carlo (MCMC) simulations were run for 10,000,000 generations with a sampling frequency of 100 and a burn-in of 25,000 trees. The remaining trees were used to generate a consensus tree and calculate posterior probabilities according to the majority rule. The topologies of phylogenetic trees were visualized by MEGA X according to Kumar et al. [54].

### Topology testing

The approximately unbiased (AU) test was used to assess the monophyly of Deviatidae and *Deviata* [55]. The constrained ML tree was enforced with the same parameters as the unconstrained ML tree, which strengthens the hypothetical relationship of the respective target taxa or group. Sitewise likelihoods for the resulting constrained and non-constrained topologies were calculated using PAUP [56] and then analyzed by CONSEL [57] to obtain *p*-values.

## Data Availability

The GenBank accession number for the new species is OR691402. One permanent slide (registration number: LLJ2020122601/1) containing the holotype specimen, circled with black ink on the back of the slide and eighteen paratype slides (registration numbers: LLJ2020122601/2–19) with protargol-stained morphostatic and dividing specimens have been deposited in the Laboratory of Systematic Taxonomy, Ocean University of China, China. One another slide with protargol-stained specimens (registration number: LLJ2020122601/20) has been deposited in the Marine Biological Museum, Chinese Academy of Sciences, Qingdao, China.

## Abbreviations

BI: Bayesian inference
bp: Base pairs
DKA: Dorsal kinety anlage
ML: Maximum likelihood
nov. sp.: Novum species
PCR: Polymerase chain reaction
SSU rRNA: Small subunit ribosomal RNA
